# Severe Immune Thrombocytopenia Following Endovascular Aneurysm Repair: A Rare Case of Helicobacter pylori Infection and Probable Systemic Lupus Erythematosus

**DOI:** 10.7759/cureus.88588

**Published:** 2025-07-23

**Authors:** Dhayananth Rattaipalivalasu Saravanan, Manogna Pendyala, Purnoor Kaur, Raheel Jamal, Mohammad Al-Nsour

**Affiliations:** 1 Internal Medicine, Mercy Health St. Vincent Medical Center, Toledo, USA; 2 Pulmonary and Critical Care Medicine, Mercy Health St. Vincent Medical Center, Toledo, USA; 3 Oncology, Mercy Health St. Vincent Medical Center, Toledo, USA

**Keywords:** autoimmune thrombocytopenia, helicobacter pylori and itp, h pylori immune response, immune thrombocytopenia, itp after evar, itp in autoimmune disease, lupus-associated itp, platelet autoantibodies, secondary itp, thrombocytopenia case report

## Abstract

We describe a rare case of a 64-year-old male who developed life-threatening thrombocytopenia two weeks after undergoing endovascular aneurysm repair (EVAR) for an abdominal aortic aneurysm. He presented with mucocutaneous bleeding manifestations, and laboratory findings revealed a critically low platelet count (<2 × 10^9^/L). An extensive workup identified a positive *Helicobacter pylori* stool antigen test and autoimmune markers, including antinuclear antibody (ANA) and anti-double-stranded DNA (anti-dsDNA) antibodies. The close temporal relationship with EVAR initially raised concern for procedure-related complications, such as heparin-induced thrombocytopenia, although this was ultimately ruled out. Given the severity and refractoriness of thrombocytopenia to steroids alone, intravenous immunoglobulin (IVIG) was added. The patient's platelet counts improved following combined corticosteroid, IVIG, and *Helicobacter pylori* eradication therapy. This unusual confluence of a recent vascular procedure, infectious, and autoimmune triggers highlights a rare and diagnostically challenging case of secondary immune thrombocytopenia (ITP), emphasizing the need for comprehensive evaluation and tailored management in similar clinical scenarios.

## Introduction

Immune thrombocytopenia (ITP) is an autoimmune disorder characterized by isolated thrombocytopenia (platelet count <100 × 10^9^/L) resulting from platelet destruction mediated by autoantibodies directed against platelet surface antigens [1]. Secondary forms of ITP are associated with various underlying conditions, including infections (notably *Helicobacter pylori*), autoimmune diseases such as systemic lupus erythematosus (SLE), and medications [2-4]. Multiple studies have highlighted the association between *Helicobacter pylori* infection and secondary ITP, suggesting that mechanisms such as molecular mimicry and immune dysregulation may be potential contributors [5]. The complexity escalates when concomitant autoimmune conditions are present, complicating the diagnosis and treatment. ITP in SLE occurs in approximately 7-30% of patients and is associated with autoantibody-mediated platelet destruction and immune complex formation, significantly complicating clinical management and therapeutic strategies [6]. This report discusses an uncommon scenario of severe secondary ITP triggered by concurrent *Helicobacter pylori* infection and autoimmune markers post-endovascular aneurysm repair (EVAR).

## Case presentation

**Figure 1:**
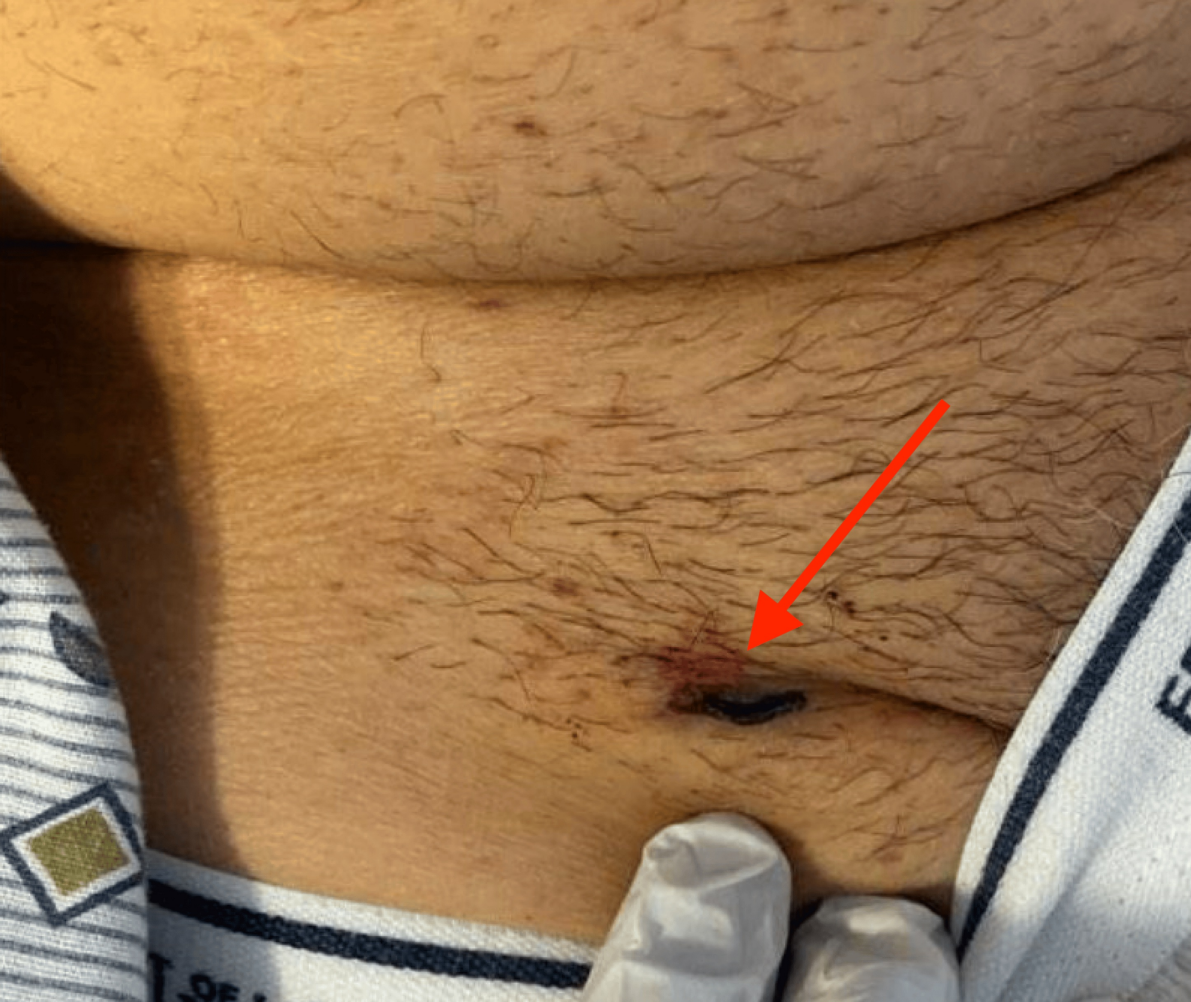
Active bleeding and clotted blood observed at the right femoral access site, as indicated by the red arrow

**Figure 2:**
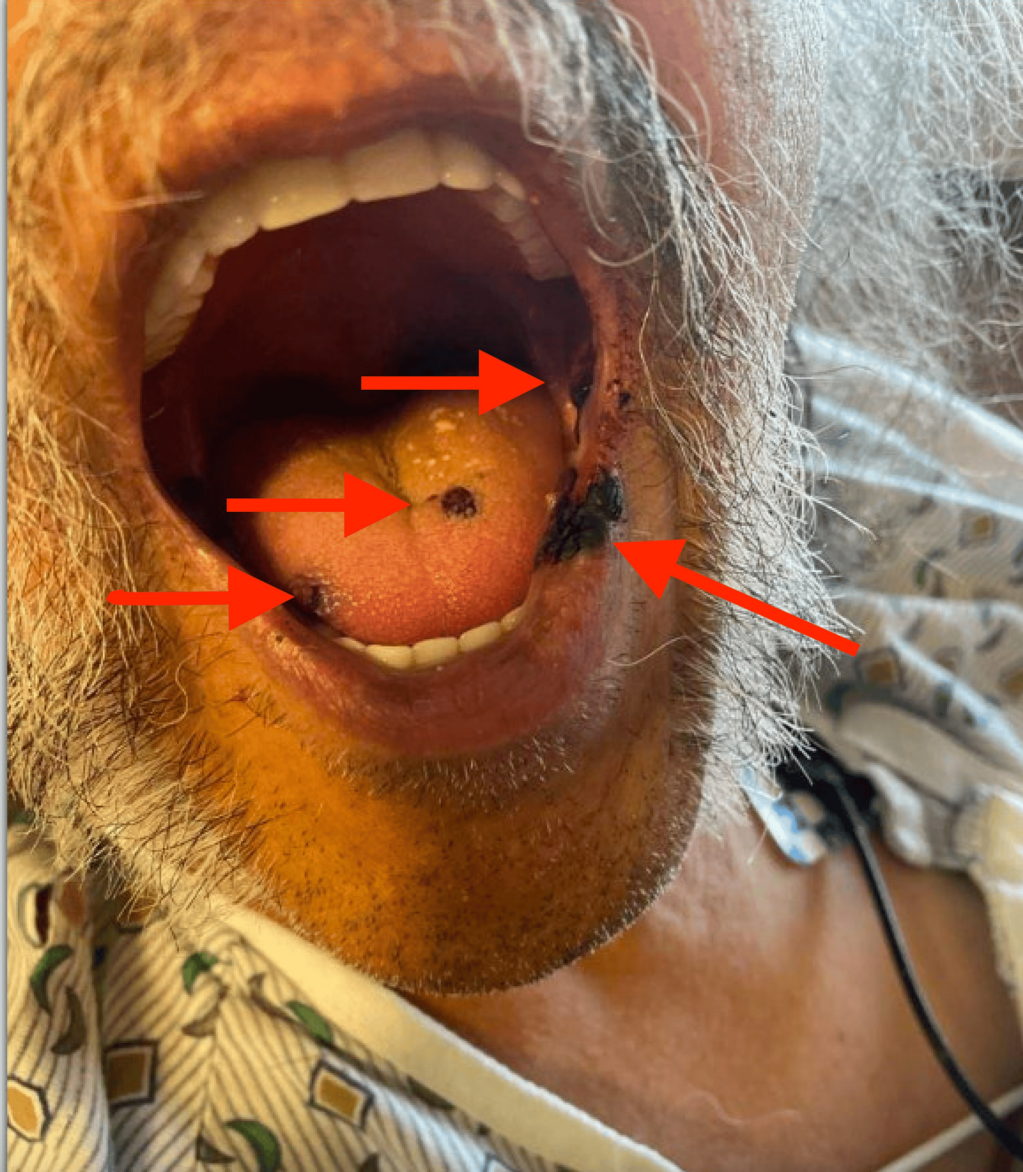
Multiple areas of oral ecchymosis were observed upon presentation, as indicated by the red arrows

A 64-year-old male with a past medical history significant for abdominal aortic aneurysm status post-EVAR, hyperlipidemia, and a 40-pack-year history of tobacco abuse (stopped six months prior) presented to the emergency department with complaints of blood-tinged discharge from his right groin dressing site and new-onset bruising in the oral cavity. These symptoms occurred two weeks after he underwent EVAR with the placement of an aortic-bi-iliac endograft for the repair of an infrarenal abdominal aortic aneurysm. The patient denied any recent trauma, gastrointestinal bleeding, hematuria, melena, hematemesis, chest pain, dyspnea, or constitutional symptoms. On initial evaluation, the patient was hemodynamically stable and saturating well on room air. Physical examination showed ecchymosis at the right groin access site with blood oozing through the dressing (Figure 1), along with multiple areas of ecchymosis noted in the oral cavity (Figure 2).

Initial laboratory studies showed severe thrombocytopenia with a platelet count <2 × 10^9^/L (reference range: 150-450 × 10^9^/L) in complete blood count (Table 1). His complete metabolic panel was unremarkable, and there was no evidence of acute kidney injury or liver dysfunction. Urinalysis revealed moderate urine hemoglobin with a clear appearance, suggestive of microscopic hematuria. Notably, the patient’s platelet count was 163 × 10^9^/L (reference range: 150-450 × 10^9^/L) post-EVAR, two weeks before presentation.

**Table 1:** Complete blood count (CBC) and related parameters at admission

Parameter	Result	Reference range	Interpretation
White blood cell count (WBC)	8.3 × 10⁹/L	4.0-10.5	Normal
Red blood cell count (RBC)	4.70 × 10¹²/L	4.2-5.9	Normal
Hemoglobin	13.6 g/dL	13.5-17.5	Normal
Hematocrit	40.6%	41-53	Low
Mean corpuscular volume (MCV)	86.4 fL	80-100	Normal
Mean corpuscular hemoglobin (MCH)	28.9 pg	27-34	Normal
Mean corpuscular hemoglobin concentration (MCHC)	33.5 g/dL	32-36	Normal
Red cell distribution width (RDW)	12.8%	11.5-14.5	Normal
Platelet count	<2 × 10⁹/L	150-450 ×10⁹/L	Critically low
Neutrophils, relative (%)	68%	40-60	High
Lymphocytes, relative (%)	19%	20-40	Low
Monocytes, relative (%)	11%	2-10	Slightly high
Eosinophils, relative (%)	0%	1-4	Low
Basophils, relative (%)	0%	<1	Normal
Neutrophils, absolute count	5.63 × 10⁹/L	1.8-7.7	Normal
Lymphocytes, absolute count	1.60 × 10⁹/L	1.0-4.8	Normal
Monocytes, absolute count	0.94 × 10⁹/L	0.1-0.8	High
Eosinophils, absolute count	<0.03 × 10⁹/L	0.0-0.5	Low
Basophils, absolute count	0.03 × 10⁹/L	0.0-0.2	Normal
Immature granulocytes, relative (%)	1%	0-0.4	High
Immature granulocytes, absolute count	0.05 × 10⁹/L	0.00-0.03	High
Nucleated red blood cells (NRBC), automated	0.0	0	Normal
Haptoglobin	381 mg/dL	30-200	High
Erythrocyte sedimentation rate (ESR)	119 mm/hour	0-20	Markedly high
Immature reticulocyte fraction (IRF)	17.0%	4-15	High
Reticulocyte percentage	0.8%	0.5-2.0	Normal
Absolute reticulocyte count	0.039 × 10⁹/L	0.025-0.075	Normal
Reticulocyte hemoglobin content	29.9 pg	28-35	Normal

Given the critically low platelet count and recent vascular intervention, he was admitted to the medical intensive care unit for close monitoring and further workup. Empiric treatment for ITP was initiated with high-dose dexamethasone (40 mg intravenous daily for four days). Due to the severity of thrombocytopenia and suboptimal initial response to steroids, intravenous immunoglobulin (IVIG) was added to the treatment regimen.

A comprehensive evaluation for secondary causes of thrombocytopenia was conducted (Table 2). This included testing for autoimmune etiologies such as antinuclear antibody (ANA) and anti-double-stranded DNA (anti-dsDNA) antibodies; infectious causes including human immunodeficiency virus (HIV), hepatitis C virus (HCV), cytomegalovirus (CMV), Epstein-Barr virus (EBV), and *Helicobacter pylori* antigen in stool; endocrine assessment with thyroid-stimulating hormone (TSH); evaluation for nutritional deficiencies including vitamin B12 and folate; hematologic conditions such as ADAMTS13 activity and serum protein electrophoresis; and a peripheral blood smear analysis. Peripheral smear analysis showed marked thrombocytopenia but otherwise unremarkable. The platelet factor 4 antibody was positive; however, the serotonin release assay was negative, effectively ruling out heparin-induced thrombocytopenia (HIT). Results also revealed positive ANA and anti-dsDNA antibodies, as well as a positive *Helicobacter pylori* stool antigen. His C-reactive protein (CRP) was elevated at 38.0 mg/L (reference range:0.0-5.0 mg/L). Other infectious and autoimmune markers were unremarkable. Based on the clinical presentation and laboratory results, a diagnosis of secondary ITP, likely triggered by *Helicobacter pylori* infection and possibly SLE, was made.

**Table 2:** Laboratory evaluation for secondary causes of immune thrombocytopenia (ITP)

Laboratory parameter	Result	Reference range
Antinuclear antibody	Positive	Negative
Anti-dsDNA (IU/mL)	35	<10.0
HIV screen	Non-reactive	Non-reactive
Hepatitis A IgM	Non-reactive	Non-reactive
Hepatitis B surface antigen	Non-reactive	Non-reactive
Hepatitis C antibody	Non-reactive	Non-reactive
Hepatitis B core antibody, IgM	Non-reactive	Non-reactive
Cytomegalovirus IgG	744	<0.5
Cytomegalovirus IgM	0.2	<0.7
Epstein-Barr virus (EBV) viral capsid antigen IgG (U/mL)	>750.0	0.0-21.9
EBV viral capsid antigen IgM (U/mL)	26.5	0.0-43.9
EBV nuclear antigen antibody IgG (U/mL)	>600.0	0.0-21.9
EBV antigen Ab, IgG (U/mL)	>150.0	0.0-10.9
*Helicobacter pylori* antigen	Positive	-
Thyroid-stimulating hormone (uIU/mL)	1.24	0.27-4.20
Vitamin B12 (pg/mL)	624	232-1245
Folate (ng/mL)	11.7	4.8-24.2
ADAMTS13 activity (%)	>100	>61%
Heparin-induced platelet antibody (optical density (OD))	1.247	0.000-0.400
Serotonin release assay	Negative	Negative

The patient was initiated on quadruple therapy for *Helicobacter pylori* eradication, consisting of bismuth subsalicylate, metronidazole, tetracycline, and pantoprazole. He continued to receive prednisone orally after completing the dexamethasone course. His platelet counts began to improve on the third day of hospitalization and continued to rise steadily with ongoing therapy (Table 3). 

**Table 3:** Platelet count trend during hospitalization

Hospital day	Platelet count (k/µL)	Interpretation
Day 1	<2	Severe thrombocytopenia
Day 2	3	Minimal improvement
Day 3	8	Gradual improvement
Day 4	6	Mild drop; still critically low
Day 5	17	Continued upward trend
Day 6 (at discharge)	29	Marked improvement

The patient remained hemodynamically stable throughout his hospitalization without the development of overt bleeding or new symptoms. He was subsequently transferred from the ICU to the internal medicine service. At the time of discharge, his platelet count had improved significantly, and he was clinically stable. At the time of discharge, his platelet count was 29000/uL. He was discharged on oral prednisone 20 mg with taper and *Helicobacter pylori* eradication therapy with plans for close outpatient follow-up with hematology and his primary care physician. At his most recent follow-up visit with hematology-oncology after four weeks from discharge, his platelet count was 172 k/uL, and he was on prednisone 5 mg; the plan was to continue taper. 

## Discussion

Secondary ITP accounts for a significant proportion of non-idiopathic thrombocytopenia cases, demanding a thorough and systematic diagnostic evaluation to identify and address underlying etiologies. In the present case, the patient experienced an acute and profound drop in platelet count within two weeks of undergoing EVAR, prompting concern for both procedure-related and systemic causes [2]. Given the nature of EVAR, which commonly involves heparin administration, it was critical to exclude HIT as a potential cause. While the initial platelet factor 4 antibody test was positive, the confirmatory serotonin release assay was negative, ruling out HIT with high specificity [7].

A key discovery in this case was the positive stool antigen for *Helicobacter pylori*. Numerous studies have demonstrated a strong association between *Helicobacter pylori* infection and the development of secondary ITP. The proposed mechanism centers on molecular mimicry, wherein antibodies generated against *Helicobacter pylori* antigens cross-react with platelet glycoproteins, leading to platelet destruction [5]. Additional mechanisms may include alterations in the host immune response, such as upregulation of Fcγ receptors on macrophages and dysregulated cytokine production [8]. Notably, eradication of *Helicobacter pylori* has been shown to result in a significant and sustained increase in platelet counts in a subset of affected patients, lending both diagnostic and therapeutic relevance to screening for the infection in cases of unexplained thrombocytopenia [3,5].

Adding further complexity to this case was the detection of autoantibodies, specifically ANA and anti-dsDNA, which raised the possibility of underlying SLE. Thrombocytopenia is one of the most common hematologic manifestations of SLE and may be mediated by autoantibodies directed against platelet surface antigens, complement-mediated lysis, and T-cell dysfunction [6,9]. Although the patient did not fulfill the clinical criteria for a definitive diagnosis of SLE, the presence of these autoantibodies suggests a predisposition or evolving autoimmune condition that may have synergistically contributed to the development of ITP. This clinical scenario suggests an interesting condition, ANA-positive primary ITP, with a potential to evolve into SLE or another connective tissue disease, as indicated by the presence of anti-dsDNA antibodies. Patients with ANA-positive primary ITP are at risk of developing severe thrombocytopenia in the postoperative period, as major abdominal surgery can act as a trigger, either unmasking previously undiagnosed ITP or exacerbating thrombocytopenia in known cases.

Management of ITP, particularly in cases involving multiple potential secondary contributors, requires a multifaceted approach. Corticosteroids are the first-line therapy and function by suppressing antibody production and reducing macrophage-mediated platelet clearance [10,11]. In patients with severe thrombocytopenia or those who do not respond adequately to steroids, IVIG serves as an effective adjunct by blocking Fc receptors on macrophages and impeding platelet destruction [11,12]. In this case, a combination of high-dose dexamethasone, IVIG, and targeted *Helicobacter pylori* eradication using quadruple therapy led to a prompt and sustained rise in platelet counts, affirming the value of integrated therapeutic strategies [10-13].

This case underscores the diagnostic challenge posed by secondary ITP, especially when overlapping factors such as recent vascular procedures, latent infections, and autoimmune markers are present. It highlights the necessity for a broad and methodical workup to avoid premature closure on a single etiology. Moreover, the therapeutic success seen in this case reinforces the importance of addressing all contributing factors simultaneously rather than sequentially.

## Conclusions

This case highlights a rare and diagnostically complex instance of secondary ITP following EVAR, driven by a multifactorial interplay of recent heparin exposure, *Helicobacter pylori* infection, and emerging autoimmune features suggestive of SLE. The exclusion of HIT and the identification of both infectious and autoimmune contributors enabled timely and targeted intervention. The patient’s favorable response to corticosteroids, IVIG, and *Helicobacter pylori* treatment underscores the value of a multifaceted approach in managing secondary ITP. This report enriches the medical literature by illustrating a rare and intricate presentation of secondary ITP arising from the convergence of procedural, infectious, and autoimmune factors. It underscores the critical need for a broad diagnostic approach, rapid recognition, and etiology-specific treatment. Such complex cases exemplify how timely, individualized management can lead to successful outcomes in high-risk hematologic conditions.

## References

[1] Rodeghiero F, Stasi R, Gernsheimer T (2009). Standardization of terminology, definitions and outcome criteria in immune thrombocytopenic purpura of adults and children: report from an international working group. Blood.

[2] Stasi R (2012). Immune thrombocytopenia: pathophysiologic and clinical update. Semin Thromb Hemost.

[3] Stasi R, Willis F, Shannon MS, Gordon-Smith EC (2009). Infectious causes of chronic immune thrombocytopenia. Hematol Oncol Clin North Am.

[4] Gasbarrini A, Franceschi F, Tartaglione R, Landolfi R, Pola P, Gasbarrini G (1998). Regression of autoimmune thrombocytopenia after eradication of Helicobacter pylori. Lancet.

[5] Franchini M, Veneri D (2006). Helicobacter pylori-associated immune thrombocytopenia. Platelets.

[6] Jung JH, Soh MS, Ahn YH, Um YJ, Jung JY, Suh CH, Kim HA (2016). Thrombocytopenia in systemic lupus erythematosus: clinical manifestations, treatment, and prognosis in 230 patients. Medicine (Baltimore).

[7] Arepally GM (2017). Heparin-induced thrombocytopenia. Blood.

[8] Kuwana M (2014). Helicobacter pylori-associated immune thrombocytopenia: clinical features and pathogenic mechanisms. World J Gastroenterol.

[9] Audia S, Mahévas M, Samson M, Godeau B, Bonnotte B (2017). Pathogenesis of immune thrombocytopenia. Autoimmun Rev.

[10] Neunert C, Terrell DR, Arnold DM (2019). American Society of Hematology 2019 guidelines for immune thrombocytopenia. Blood Adv.

[11] Provan D, Arnold DM, Bussel JB (2019). Updated international consensus report on the investigation and management of primary immune thrombocytopenia. Blood Adv.

[12] Matzdorff A, Meyer O, Ostermann H (2018). Immune thrombocytopenia - current diagnostics and therapy: recommendations of a joint working group of DGHO, ÖGHO, SGH, GPOH, and DGTI. Oncol Res Treat.

[13] Rostami N, Keshtkar-Jahromi M, Rahnavardi M, Keshtkar-Jahromi M, Esfahani FS (2008). Effect of eradication of Helicobacter pylori on platelet recovery in patients with chronic idiopathic thrombocytopenic purpura: a controlled trial. Am J Hematol.

